# Durability of Antibody Response and Frequency of SARS-CoV-2 Infection 6 Months after COVID-19 Vaccination in Healthcare Workers

**DOI:** 10.3201/eid2804.212037

**Published:** 2022-04

**Authors:** Eric D. Laing, Carol D. Weiss, Emily C. Samuels, Si’Ana A. Coggins, Wei Wang, Richard Wang, Russell Vassell, Spencer L. Sterling, Marana S. Tso, Tonia Conner, Emilie Goguet, Matthew Moser, Belinda M. Jackson-Thompson, Luca Illinik, Julian Davies, Orlando Ortega, Edward Parmelee, Monique Hollis-Perry, Santina E. Maiolatesi, Gregory Wang, Kathleen F. Ramsey, Anatalio E. Reyes, Yolanda Alcorta, Mimi A. Wong, Alyssa R. Lindrose, Christopher A. Duplessis, David R. Tribble, Allison M.W. Malloy, Timothy H. Burgess, Simon D. Pollett, Cara H. Olsen, Christopher C. Broder, Edward Mitre

**Affiliations:** Uniformed Services University, Bethesda, Maryland, USA (E.D. Laing, E.C. Samuels, S.A. Coggins, S.L. Sterling, M.S. Tso, T. Conner, E. Goguet, M. Moser, B.M. Jackson-Thompson, L. Illinik, J. Davies, O. Ortega, E. Parmelee, A.R. Lindrose, D.R. Tribble, A.M.W. Malloy, T.H. Burgess, S.D. Pollett, C.H. Olsen, C.C. Broder, E. Mitre);; US Food and Drug Administration, Silver Spring, Maryland, USA (C.D. Weiss, W. Wang, R. Wang, R. Vassell);; Henry M. Jackson Foundation for the Advancement of Military Medicine, Inc., Bethesda (E.C. Samuels, S.A. Coggins, S.L. Sterling, E. Goguet, M. Moser, B.M. Jackson-Thompson, L. Illinik, J. Davies, O. Ortega, E. Parmelee, S.E. Maiolatesi, A.R. Lindrose, S.D. Pollett);; Naval Medical Research Center, Silver Spring (L. Illinik, J. Davies, O. Ortega, E. Parmelee, M. Hollis-Perry, S.E. Maiolatesi, G. Wang, K.F. Ramsey, A.E. Reyes, Y. Alcorta, M.A. Wong, C.A. Duplessis);; General Dynamics Information Technology, Falls Church, Virginia, USA (G. Wang, K.F. Ramsey, A.E. Reyes, Y. Alcorta, M.A. Wong)

**Keywords:** COVID-19, coronavirus disease, SARS-CoV-2, severe acute respiratory syndrome coronavirus 2, viruses, respiratory infections, zoonoses, vaccine-preventable diseases, BNT162b2, healthcare workers, United States

## Abstract

Severe acute respiratory syndrome coronavirus 2 (SARS-CoV-2) antibodies decay but persist 6 months postvaccination; lower levels of neutralizing titers persist against Delta than wild-type virus. Of 227 vaccinated healthcare workers tested, only 2 experienced outpatient symptomatic breakthrough infections, despite 59/227 exhibiting serologic evidence of SARS-CoV-2 infection, defined as presence of nucleocapsid protein antibodies.

Neutralizing antibodies (nAbs) and binding antibodies (bAbs) appear to be associated with protection against symptomatic severe acute respiratory syndrome coronavirus 2 (SARS-CoV-2) infection and coronavirus disease (COVID-19) (*1**,**2*). Early assessments of the Pfizer-BioNTech (https://www.pfizer.com) BNT162b2 COVID-19 mRNA vaccine observed >95% effectiveness against predominantly Alpha infections (*3*), but the potential effect of waning postvaccine neutralizing titers is an ongoing concern (*4*).

Apparent increases in vaccine-breakthrough infections may result from waning antibody titers, increases in exposure risk, and reduced vaccine effectiveness against Delta and other variants. In mid-2021, Delta became the dominant virus type in the United States (*5*). Delta appears to cause increased hospitalization rates and has increased transmissibility compared with Alpha and other pre-Delta variants (*6*; Bolze et al., unpub. data, https://doi.org/10.1101/2021.06.20.21259195). We report bAb and nAb levels as well as clinically overt and asymptomatic breakthrough infections that occurred among US healthcare workers in the Prospective Assessment of SARS-CoV-2 Seroconversion (PASS) study (*7*), conducted during January–August 2021.

## The Study

The PASS study protocol was approved by the Uniformed Services University of the Health Sciences Institutional Review Board (Federalwide Assurance no. 00001628, US Department of Defense Assurance no. P60001) in compliance with all applicable federal regulations governing the protection of human participants. Written consent was obtained from all study participants.

For the PASS study, we enrolled and followed generally healthy, adult healthcare workers (HCWs) at Walter Reed National Military Medical Center (Bethesda, MD, USA) who were seronegative for IgG to SARS-CoV-2 spike glycoprotein (spike) and had no history of COVID-19, as previously described (*7*). We collected participants’ serum samples monthly and screened them for IgG against SARS-CoV-2 spike and nucleocapsid protein (NP) in multiplex microsphere-based immunoassays, as previously described (Appendix) (E.D. Laing, unpub. data, ). In addition, we asked participants to obtain nasopharyngeal SARS-CoV-2 PCR testing at a designated COVID-19 testing center if they experienced symptoms consistent with SARS-CoV-2 infection.

To quantify spike IgG bAbs in World Health Organization binding antibody units (BAU), we interpolated IgG levels against an internal standard curve calibrated to the Human SARS-CoV-2 Serology Standard (Appendix Figure 1). We assessed serum samples for nAbs against SARS-CoV-2 wild type and Delta as previously described by using a well-characterized SARS-CoV-2 lentiviral-pseudovirus neutralization assay (Appendix) (*8*). 

**Figure 1:**
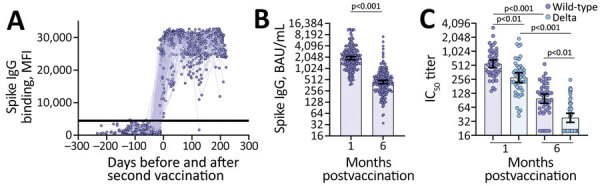
Vaccine-induced binding and neutralizing antibody responses observed among US healthcare worker participants in the Prospective Assessment of SARS-CoV-2 Seroconversion (PASS) study, January–August 2021. A) MFI levels of vaccine-induced spike IgG binding before and after second vaccination in serum samples diluted 1:400 (n = 227 participants). Horizontal line indicates the positive or negative spike IgG threshold. B) Spike IgG binding antibodies (BAU/mL) quantified from serum samples collected 1 month (mean 36.9 days, range 23–81 days) and 6 months (mean 201.1 days, range 151–237 days) postvaccination (n = 187 participants). Wilcoxon matched-pairs signed rank test performed; y-axis is log_2_-scale. C) Neutralizing antibody titers against severe acute respiratory syndrome coronavirus 2 wild-type and Delta variant from serum samples collected 1 month (mean 30.8 days, range 28–42 days) and 6 months (mean 200.1 days, range 189–219 days) postvaccination (n = 49 participants). Friedman ANOVA with Dunn’s multiple comparisons performed post-hoc; y-axis is log_2_-scale. All errors bars represent the geometric mean and 95% CIs. BAU, binding antibody units; IC_50_, 50% inhibitory concentration; MFI, median fluorescence intensity.

Excluding persons infected before January 31, 2021, the study followed 227 participants fully vaccinated with BNT162b2 vaccine and 17 unvaccinated participants. Participants were generally healthy, had a mean age of 41.7 (range 20–69) years, and were predominantly women (Table). Vaccinated and unvaccinated participants reported similar in-hospital time; >70% of each group worked in the hospital >15 days per month, and had similar rates of direct interaction with COVID-19 positive patients (monthly average of 47% for vaccinated and 45% for unvaccinated participants).

**Table:** Demographic characteristics of US healthcare worker participants in the Prospective Assessment of SARS-CoV-2 Seroconversion (PASS) study, January–August 2021*

Characteristic	BNT162b2 vaccinated	Vaccinated with 6-mo follow-up bAb	Vaccinated with 6-mo follow-up nAb titers	Unvaccinated
Total	227 (100)	187 (100)	49 (100)	17 (100)
Sex				
F	159 (70)	131 (70)	33 (67)	13 (76)
M	68 (30)	56 (30)	16 (33)	4 (24)
Ethnicity				
Non-Hispanic	209 (92)	175 (94)	47 (96)	16 (94)
Hispanic	14 (6)	10 (5)	1 (2)	1 (6)
Not reported	4 (2)	2 (1)	1 (2)	0
Race				
White	165 (73)	139 (74)	35 (71)	9 (53)
Black	26 (11.5)	18 (9.5)	4 (8)	6 (35)
Asian	23 (10)	19 (10)	8 (16)	1 (6)
>2 races	7 (3)	6 (3)	1 (2)	1 (6)
Native Hawaiian or other Pacific Islander	1 (0.5)	1 (0.5)	1 (2)	0
Not reported	5 (2)	4 (2)	0	0
Age, y, mean (range)	41.7 (20–69)	42.8 (21–69)	44.7 (26–69)	32.3 (19–50)

*Values are no. (%) except as indicated. bAb, binding antibodies; nAb, neutralizing antibodies.

We observed seroconversion in all participants 1 month after the second vaccine dose (Figure 1, panel A). We quantified spike IgG bAbs at 1 and 6 months after full vaccination in the 187 vaccinated participants with serum samples collected at both timepoints. Spike IgG bAbs decreased from a geometric mean of 1,929 BAU/mL (95% CI 1,752–2,124 BAU/mL) at 1 month postvaccination to a geometric mean of 442 BAU/mL (95% CI 399–490 BAU/mL) at 6 months postvaccination (p<0.001) (Figure 1, panel B). Similarly, we observed decay of nAbs between the 1- and 6-month postvaccination timepoints. Peak SARS-CoV-2 wildtype nAbs decreased from a geometric mean titer (GMT) of 551 (95% CI 455–669 GMT) to 98 GMT (95% CI 78–124 GMT) 6 months after full vaccination (Figure 1, panel C). The GMTs of nAbs were significantly higher against wild-type compared with Delta SARS-CoV-2 at both timepoints after vaccination (Figure 1, panel C). In comparison, nAbs against Delta decayed from 279 GMT (95% CI 219–355 GMT) at peak to 38 GMT (95% CI 31–48 GMT) after 6 months. Quantitative IgG bAb (in BAU/mL) correlated with nAb titers (ρ = 0.70; p<0.001), demonstrating comparable decay of IgG bAbs and nAbs (Appendix Figure 2).

**Figure 2:**
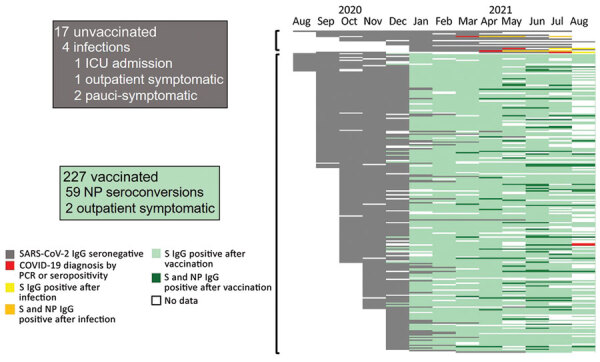
Timeline of antibody responses and SARS-CoV-2 infections among US healthcare worker participants in the Prospective Assessment of SARS-CoV-2 Seroconversion (PASS) study, January–August 2021 (17 unvaccinated and 227 vaccinated participants). Each horizontal bar represents the infection, vaccination, and serologic status obtained monthly in all participants who had not been diagnosed with SARS-CoV-2 by PCR or S protein IgG seroconversion by January 31, 2021. White spaces indicate no data. Gray bars represent negative S protein IgG. Red bars indicate month of SARS-CoV-2 diagnosis by PCR positivity or S protein IgG seroconversion. Yellow bars indicate S protein IgG seroconversion after SARS-CoV-2 diagnosis in unvaccinated persons, and orange bars indicate presence of both S protein and NP antibodies in unvaccinated persons. Light green bars indicate S protein IgG seroconversion after vaccination. Dark green bars indicate detection of NP IgG in addition to S protein antibodies at timepoints postvaccination. S, spike protein; NP, nucleocapsid protein; ICU, intensive care unit; SARS-CoV-2, severe acute respiratory syndrome coronavirus 2.

In addition to spike IgG bAbs, we also monitored for seroconversion of IgG bAbs to NP. Of vaccinated participants, 26.0% (59/227) had NP seroconversion during March–August 2021 (Figure 2). Only 2 had symptomatic, PCR-positive, vaccine-breakthrough infections, both of which were self-limited, outpatient cases. In the unvaccinated cohort, 4 participants had SARS-CoV-2 infection diagnosed: 2 by PCR while experiencing symptomatic infection (1 outpatient case, 1 requiring intensive care) and 2 diagnosed by spike IgG seroconversion and who reported mild symptoms retrospectively. The frequency of NP seroconversions in the vaccinated population correlated with the frequency of SARS-CoV-2 infections diagnosed in the unvaccinated participants (23.5% [4/17]) (Figure 2), suggesting similar exposure rates.

## Conclusions

In this prospective cohort study of generally healthy, adult HCWs, we found that SARS-CoV-2 spike IgG bAbs and nAbs induced by BNT162b2 mRNA COVID-19 vaccination wane but remained detectable through 6 months after vaccination, corroborating results of another study (*9*). Consistent with another report (*10*), we observed significantly lower vaccine-induced nAb titers against Delta compared to wild-type virus. Asymptomatic infections determined by NP seroconversions were relatively frequent, but symptomatic infection was rare, and severe disease was absent.

We observed 1 of 17 unvaccinated persons have onset of severe COVID-19, versus no severe cases among 227 vaccinated participants. Of vaccinated persons, 2 had symptomatic, PCR-proven breakthrough infections, both of which were managed as outpatient cases. We observed that 26% of vaccinated participants developed antibodies against SARS-CoV-2 NP, suggesting that vaccinated persons experienced exposures to SARS-CoV-2 as frequently as the unvaccinated population, yet rarely had onset of overt clinical disease. 

The strengths of the study include frequency of serologic assessments and use of variant specific nAb in addition to multiplexed antigen-specific IgG detection. Use of longitudinal serologic assessments (in addition to PCR testing when participants exhibited symptoms) enabled detection of asymptomatic and pauci-symptomatic SARS-CoV-2 exposures. Although our study was powered to show clear differences in antibody titers over time, limitations include the moderate size of the cohort and the small number of unvaccinated participants. Further, seasonal human coronavirus (HCoV) infections may drive cross-reactive IgG responses against SARS-CoV-2 NP. We mitigated the likelihood of HCoV-driven false-positives by using convalescent serum samples from persons with PCR-confirmed HCoV infections to establish the threshold for SARS-CoV-2 NP IgG positivity, which had a specificity of 94% in our multiplex assay (E.D. Laing et al., unpub. data). In a separate study, NP seroconversion reportedly occurred in only 71% of PCR-confirmed vaccine-breakthrough infections (*11*); thus, some instances of asymptomatic vaccine-breakthrough infections may have gone unnoticed.

We observed persistence of nAb titers against SARS-CoV-2 wild-type equal to or greater than the lowest dilution tested in 90% (44/49) of healthy adults 6 months after vaccination with BNT162b2. Neutralizing activity against Delta virus was lower,; only 47% (23/49) of participants maintained nAb titers above the lowest dilution at 6 months postvaccination. The decrease in nAb does not necessarily mean that persons have lost protection against severe COVID-19, however, given that nAb titers required for protection remain unknown and virus neutralization is only 1 function of antibodies. In addition, memory B cells and T cells have been detected 8–12 months after SARS-CoV-2 infection, demonstrating that adaptive immune memory can be long-lasting (*12*,*13*). Further research is needed to understand the correlates of protection against moderate to severe COVID-19 for known and emerging SARS-CoV-2 variants. Even so, our results suggest that the BNT162b2 vaccine confers protection against severe clinical disease caused by the variants circulating in the United States through August 2021 for >6 months in generally healthy adults, even in the face of frequent exposures to the virus and waning antibody titers.

Appendix Additional information about durability of antibody response and frequency of SARS-CoV-2 infection 6 months after COVID-19 vaccination in healthcare workers.
