# Supplementary material for: Durability of Antibody Response and Frequency of SARS-CoV-2 Infection 6 Months after COVID-19 Vaccination in Healthcare Workers
Source: Emerg Infect Dis. 2022 Apr;28(4):828–32. doi: 10.3201/eid2804.212037 (PMC8962883; doi:10.3201/eid2804.212037)
Supplement: Appendix — Additional information about durability of antibody response and frequency of SARS-CoV-2 infection 6 months after COVID-19 vaccination in healthcare workers. [file 21-2037-Techapp-s1.pdf]

Article DOI: https://doi.org/10.3201/eid2804.212037

Durability of Antibody Response and Frequency of SARS-CoV-2 Infection 6 Months after COVID-19 Vaccination in Healthcare Workers

Appendix

Methods

Multiplex Microsphere-based Immunoassay Screening Procedures

Prefusion stabilized spike (S) glycoprotein ectodomain trimers (S-2P) (*1*,*2*), hereafter referred to as spike, of SARS-CoV-2, HCoV-229E, and HCoV-NL63 were purchased from LakePharma, Inc (Hopkinton, MA, USA). HCoV-OC43 and HCoV-HKU1 spike were provided by Dr. Dominic Esposito, National Cancer Institute Frederick National Laboratory (NCI FNL), Protein Expression Laboratory, and have been previously described (*3*). A SARS-CoV-2 NP was sourced from RayBiotech (Peachtree Corners, GA, USA). Multiplexed antigen-based antibody detection has been described previously (E.D. Laing et al., unpub. data, https://doi.org/10.1101/2021.02.10.21251518; E.D. Laing et al., unpub. data, https://doi.org/10.1101/2021.04.27.21256207). Briefly, SARS-CoV-2 spike and NP, and HCoV spike were coupled to magnetic microspheres (Bio-Rad, Hercules, CA, USA). Serum samples were collected from venipuncture in serum separator tubes, processed and stored at −80°C in 500 µL aliquots until use. For weekly screening, neat human serum samples (1.25 µL) were diluted 1:400 in 1X PBS and heat inactivated at 60°C for 30 min after dilutions. Diluted serum samples were incubated with a master mix of SARS-CoV-2, HCoV-OC43, HCoV-HKU1, HCoV-229E, HCoV-NL63 spike, and SARS-CoV-2 NP coupled microspheres. This multiplex microsphere-based immunoassay has a 94% sensitivity and 100% specificity to detect anti-SARS-CoV-2 spike IgG; and a 93% sensitivity and 94% specificity to detect anti-SARS-CoV-2 NP IgG seroconversion between 7–28 days post-symptom onset (E.D. Laing et al., unpub. data, https://doi.org/10.1101/2021.02.10.21251518) (*4*,*5*).

After a 45 minute incubation of diluted serum and antigen-coupled microspheres, with agitation (900 rpm), plates were washed with PBS-Tween20 (0.05%) and 100 µL of biotinylated cross-absorbed anti-human IgG (Thermo Fisher Scientific, Waltham, MA) diluted in 1X PBS-T (1:5000) was added to each well, and plates were incubated for 45 minutes with agitation. Lastly, after washing, streptavidin-phycoerythrin was diluted 1:1000 in PBS-T, and 100 µL were added to each well and plates were incubated for 45 min with agitation (900 rpm). Plates were washed, and microspheres were resuspended with 100 µL PBS-T per well then analyzed on Bio-Plex 200 multiplexing systems (Bio-Rad) and median fluorescence intensity (MFI) values for samples are reported as the PBS adjusted average from duplicate plates. Antibody testing was blind to descriptive data, including demographic data, SARS-CoV-2 PCR status, and clinical phenotype.

Calibration to NCI FNL U.S. Serology Standard and interpolation of Binding Antibody Units (BAU/mL)

An internal reference standard (IR-std), a mixture of nine PASS study serum samples obtained 1 month after PCR-confirmed SARS-CoV-2 infection in 2020, was calibrated against the NCI FNL U.S. serology national standard (U.S.-std) for SARS-CoV-2 spike protein and NP IgG. The IR-std and U.S.-std were diluted 2-fold starting at 1:400 through 1:512,000, and IgG was detected as described above. The concentration of spike-specific IgG in IR-std was determined to be 428 BAU/ml by averaging the results of four separate analyses interpolating IR-std against a standard curve of the U.S.-std with known concentration of 764 BAU/ml. With the established IR-std BAU/mL, PASS participant serum samples were tested at 1:400 and 1:8000 dilutions. All MFI values were adjusted to the PBS-blank control wells, then MFI values were interpolated against the IR-std included on each 96-well microtiter plate. Spike IgG BAU/mL were log10-transformed, checked for normality, and statistical significance was determined by two-tailed Wilcoxon matched-pairs signed rank test.

SARS-CoV-2 S-Pseudovirus Production and Neutralization

A codon-optimized spike gene corresponding to the Wuhan-1 spike with the D614G substitution was used to make the wild-type (WT) pseudovirus. A codon-optimized spike gene used to make the Delta (B.1.617.2) pseudovirus had the following mutations on the WT backbone: T19R, G142D, E156 deletion, F157 deletion, R158G, L452R, T478K, D614G, P681R, and D950N. Neutralization assay were performed as previously described (*6*,*7*). Briefly, 5µg of pCMVΔR8.2, 5µg of pHR'CMVLuc and 0.5µg of S expression plasmids were co-

transfected in 293T cells. Pseudovirus supernatants were collected ≈48 hours post-transfection, filtered through a 0.45 µm low protein binding filter, and used immediately or stored at −80°C. Pseudovirus titers were measured by infecting 293T-ACE2.TMPRSS2 cells, which stably express human angiotensin converting enzyme 2 (ACE2) and transmembrane serine protease 2 (TMPRSS2), for 48 hours before measuring luciferase activity (luciferase assay reagent, Promega, Madison, WI). Neutralization titers were calculated using a nonlinear regression curve fit (GraphPad Prism software Inc., La Jolla, CA) using 8-point dilution curves. The mean titer from at least two independent experiments each with intra-assay duplicates was reported as the final titer. Titers below the lowest serum dilution of 1:40 were treated as 20 for statistical analysis. nAb IC_{50} titers were log10-transfomed, checked for normality, and statistical significance was determined by Friedman ANOVA with Dunn's multiple comparisons performed post-hoc.

References

1. Wrapp D, Wang N, Corbett KS, Goldsmith JA, Hsieh CL, Abiona O, et al. Cryo-EM structure of the 2019-nCoV spike in the prefusion conformation. Science. 2020;367:1260–3. PubMed https://doi.org/10.1126/science.abb2507

2. Esposito D, Mehalko J, Drew M, Snead K, Wall V, Taylor T, et al. Optimizing high-yield production of SARS-CoV-2 soluble spike trimers for serology assays. Protein Expr Purif. 2020;174:105686. PubMed https://doi.org/10.1016/j.pep.2020.105686

3. Hicks J, Klumpp-Thomas C, Kalish H, Shunmugavel A, Mehalko J, Denson JP, et al. Serologic cross-reactivity of SARS-CoV-2 with endemic and seasonal betacoronaviruses. J Clin Immunol. 2021;41:906–13. PubMed https://doi.org/10.1007/s10875-021-00997-6

4. Clifton GT, Pati R, Krammer F, Laing ED, Broder CC, Mendu DR, et al. SARS-CoV-2 infection risk among active duty military members deployed to a field hospital—New York City, April 2020. MMWR Morb Mortal Wkly Rep. 2021;70:308–11. PubMed https://doi.org/10.15585/mmwr.mm7009a3

5. Ramos I, Goforth C, Soares-Schanoski A, Weir DL, Samuels EC, Phogat S, et al. Antibody responses to SARS-CoV-2 following an outbreak among Marine recruits with asymptomatic or mild infection. Front Immunol. 2021;12:681586. PubMed https://doi.org/10.3389/fimmu.2021.681586

6. Neerukonda SN, Vassell R, Lusvarghi S, Wang R, Echegaray F, Bentley L, et al. SARS-CoV-2 delta variant displays moderate resistance to neutralizing antibodies and spike protein properties of higher soluble ACE2 sensitivity, enhanced cleavage and fusogenic activity. Viruses. 2021;13:2485. PubMed https://doi.org/10.3390/v13122485

7. Neerukonda SN, Vassell R, Herrup R, Liu S, Wang T, Takeda K, et al. Establishment of a well-characterized SARS-CoV-2 lentiviral pseudovirus neutralization assay using 293T cells with stable expression of ACE2 and TMPRSS2. PLoS One. 2021;16:e0248348. PubMed https://doi.org/10.1371/journal.pone.0248348

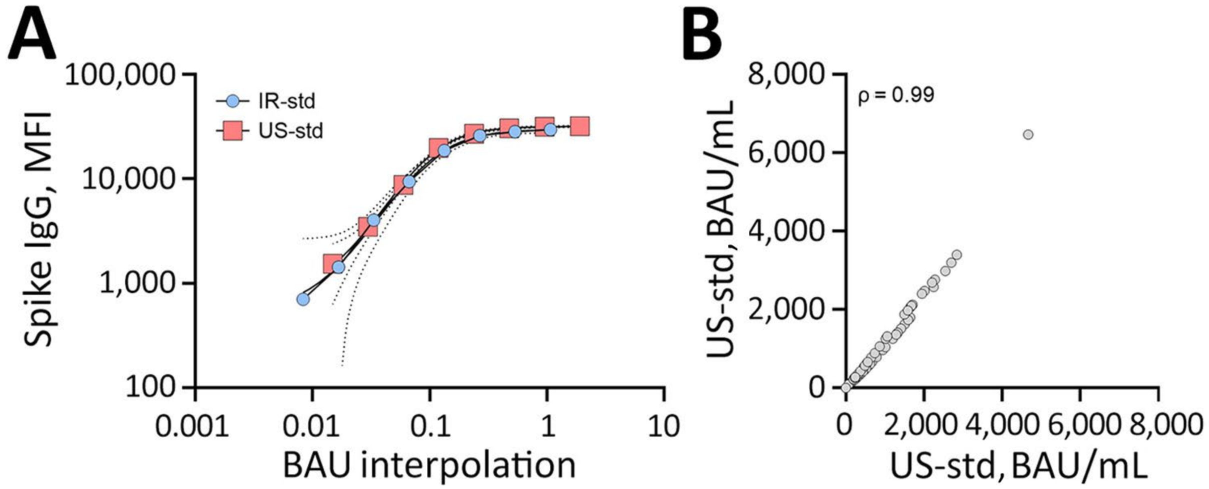

Appendix Figure 1. Comparison of the PASS internal reference standard (IR-std) to the NCI FNL U.S. serology national standard (U.S.-std). A) Comparison of the PASS internal reference standard (IR-std) curve to the NCI FNL U.S. serology national standard (U.S.-std) curve for SARS-CoV-2 spike protein reactive immunoglobulin G (IgG); MFI, median fluorescence intensity; BAU, binding antibody units; curves with dashed lines represent the mean and error bars of independent experiments, axes are log10-scale,representative of four independent experiments. B) Correlation between spike IgG BAU/mL interpolated from the IR-std or the U.S.-std; n = 76 serum samples, Spearman's rho (ρ) = 0.99, two-tailed $p < 0.001$.

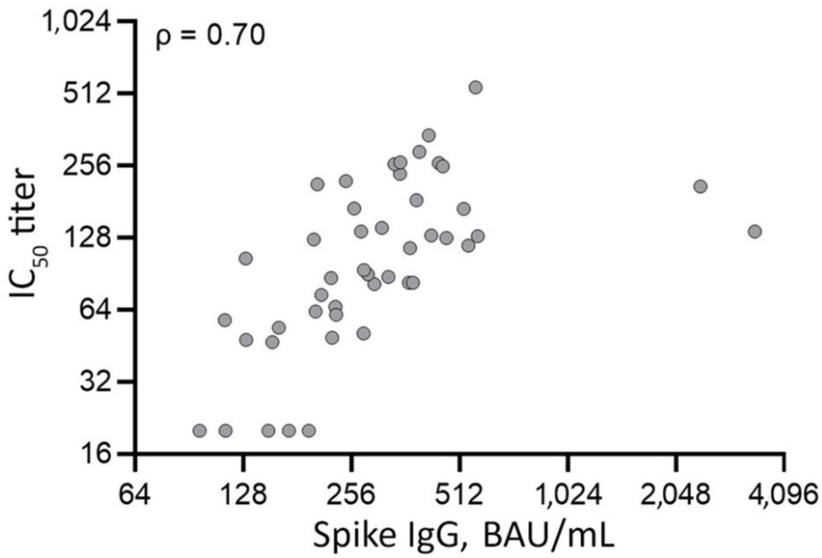

Appendix Figure 2. Correlation between spike IgG bAb and nAb titers against SARS-CoV-2 WT 6 months-post vaccination. Six months post-vaccination serum antibodies were evaluated for correlation between spike IgG bAb, and nAb IC_{50} titers against SARS-CoV-2 WT, n = 49. Spearman's rho (ρ) = 0.70, two-tailed $p < 0.001$; axes are log2-scale.
